# Arbuscular mycorrhizal fungi influence the intraspecific competitive ability of plants under field and glasshouse conditions

**DOI:** 10.1007/s00425-023-04214-z

**Published:** 2023-08-03

**Authors:** Karin Groten, Felipe Yon, Ian T. Baldwin

**Affiliations:** 1 Max Planck Institute for Chemical Ecology, Hans-Knoell-Str. 8, 07745 Jena, Germany; 2 Present Address: Instituto de Medicina Tropical, Universidad Peruana Cayetano Heredia, Lima, Peru

**Keywords:** Calcium- and calmodulin-dependent protein kinase, Field experiments, Microcosms, Monocultures, Plant communities, Plant fitness

## Abstract

**Main conclusion:**

*Nicotiana attenuata’s* capacity to interact with arbuscular mycorrhizal fungi influences its intraspecific competitive ability under field and glasshouse conditions, but not its overall community productivity.

**Abstract:**

Arbuscular mycorrhizal (AM) fungi can alter the nutrient status and growth of plants, and they can also affect plant–plant, plant–herbivore, and plant–pathogen interactions. These AM effects are rarely studied in populations under natural conditions due to the limitation of non-mycorrhizal controls. Here we used a genetic approach, establishing field and glasshouse communities of AM-harboring *Nicotiana attenuata* empty vector (EV) plants and isogenic plants silenced in calcium- and calmodulin-dependent protein kinase expression (ir*CCaMK*), and unable to establish AM symbioses. Performance and growth were quantified in communities of the same (monocultures) or different genotypes (mixed cultures) and both field and glasshouse experiments returned similar responses. In mixed cultures, AM-harboring EV plants attained greater stalk lengths, shoot and root biomasses, clearly out-competing the AM fungal-deficient ir*CCaMK* plants, while in monocultures, both genotypes grew similarly. Competitive ability was also reflected in reproductive traits: EV plants in mixed cultures outperformed ir*CCaMK* plants. When grown in monocultures, the two genotypes did not differ in reproductive performance, though total leaf N and P contents were significantly lower independent of the community type. Plant productivity in terms of growth and seed production at the community level did not differ, while leaf nutrient content of phosphorus and nitrogen depended on the community type. We infer that AM symbioses drastically increase *N. attenuata*’s competitive ability in mixed communities resulting in increased fitness for the individuals harboring AM without a net gain for the community.

**Supplementary Information:**

The online version contains supplementary material available at 10.1007/s00425-023-04214-z.

## Introduction

Interactions between plants of the same or different genotype(s) are influenced by their microbial partners, in particular, arbuscular mycorrhizal (AM) fungi belonging to the subphylum Glomeromycotina (Spatafora et al. 2016). This plant–AM fungal interaction is thought to be based on an exchange of nutrients. Due to their thin hyphae and extensively branched networks, AM fungi improve a host plant’s acquisition of fitness-limiting mineral nutrients, in particular phosphorus [up to 90% of its total phosphorous (P) uptake (Smith and Read 2008)], but also other minerals such as nitrogen (N), manganese, potassium, magnesium, zinc, and water (González-Guerrero et al. 2016; Garcia et al. 2016; Wipf et al. 2019). The plants provide up to 20% of their photoassimilates in the form of carbohydrates (Smith and Read 2008) and lipids to the fungus (Bravo et al. 2017). Mycorrhiza-specific phosphate transporters are induced during fungal colonization, enabling an indirect route of P uptake that complements direct P uptake via roots (Smith et al. 2004). Furthermore, recent studies show that not only active inoculum but also the AM fungal necromass increases plant growth (Jansa et al. 2020). AM fungi can also improve a plant’s resistance against biotic and abiotic stressors, such as drought, heat, pathogen or herbivore attack (Santander et al. 2017; Rivero et al. 2018; Sanmartin et al. 2020), providing additional routes to altered plant performance from the interaction.

The vast majority of land plants interact with AM fungi (Brundrett and Tedersoo 2018). The earliest described interaction dates to more than 400 million years ago (Strullu-Derrien et al. 2018), and the interaction may have been crucial for the colonization of land by plants (Brundrett and Tedersoo 2018). The molecular dialogue between plants and AM fungi starts before their physical interaction and is governed by a conserved set of genes for fungal recognition and signaling that are required for the establishment of a functional symbiosis (e.g., MacLean et al. 2017; Wipf et al. 2019; Xue and Wang 2020). One key element is a calcium- and calmodulin-dependent protein kinase (CCaMK) located in the nucleus and thought to decode a Ca^2+^ signal to establish a complex with IPD3 (INTERACTING PROTEIN OF DOES NOT MAKE INFECTIONS 3, MacLean et al. 2017). Plants impaired in the expression of *CCaMK* do not establish a functional symbiosis (Levy et al. 2004).

The costs/benefits of AM fungal inoculation for a host plant are commonly evaluated by growing plants with and without AM fungi to calculate the differences in growth and fitness, such as biomass, flower and seed numbers, between inoculated and non-inoculated plants (Chaudhary et al. 2016). The difference is also termed “response” or “responsiveness” (Wilson and Hartnett 1998; Konvalinková and Jansa 2016) and many studies have revealed a large variation in the response of plants, with outcomes not always positive, but neutral or negative, depending on the environment (Johnson 2010). Environmental stress factors (low light, high P), but also the plant and fungal genotype, can determine if harboring AM fungi is beneficial for plants (Berger and Gutjahr 2021; Bennett and Groten 2022). Differences in response are not only found at the species level, but also within the same species. Natural plant populations consist of many different genotypes, often in the same area. These genotypes may vary in their response to AM fungal colonization as shown for many crop varieties, e.g., wheat, barley, maize (Sawers et al. 2017; Elliott et al. 2020; Thirkell et al. 2020). For example, for maize, it was shown that different mycorrhiza-associated maize lines vary in P acquisition efficiency and in their expression of P-transporter genes (Sawers et al. 2017).

In addition to these direct effects at the individual plant level, AM fungi may alter plant–plant competition and facilitation of niche partition (Bever et al. 2012; Hodge and Fitter 2013). AM fungi colonize more than 72–80% of all land plant species (Brundrett and Tedersoo 2018). Their fungal external hyphae spread out in the surrounding soil and can colonize neighboring plants of the same or different species at the same time, thus creating a so-called common mycorrhizal network (CMN) (Barto et al. 2012). The CMN may modify the distribution of limiting mineral nutrients among plants (Lekberg et al. 2010; Weremijewicz et al. 2016), but also exchange information, e.g., about pathogen or herbivore attack of neighboring plants (Babikova et al. 2014; Song et al. 2014, 2019). In vitro experiments have revealed that AM fungi move P from P-rich to P-poor nutrient patches (Whiteside et al. 2019). Differences in sink strengths and changes in nutrient transfer belowground may influence the symmetry of competition among networked plants (Montesinos-Navarro et al. 2018; Whiteside et al. 2019; van’t Padje et al. 2021). It is assumed that under limiting conditions, plants of different species are more likely to require different resources than those of the same species (Mayfield and Levine 2010), and different plant species may differ in the efficiency with which they interact with the same fungal partner (van’t Padje et al. 2021), thus reducing competition. If an individual in a population or a species in a diverse community performs better in terms of growth and fitness than its neighbors, it may alter the plant community dynamics (Bray et al. 2003), plant diversity, and/or ecosystem productivity (van der Heijden et al. 2015). Over time, these differences in response can increase or decrease the abundance of plants. Thus, AM fungi may alter the structure of plant communities (Bennett and Cahill Jr 2016; Tedersoo et al. 2020).

A number of studies investigated how resource distributions and plant–plant competition are modified when different plant species are connected to the same CMN (Walder et al. 2012; Weremijewicz and Janos 2013; Milkereit et al. 2018). For example, AM fungal inoculation enhanced the competitive ability of perennial species competing with annual species (Lin et al. 2015). Using isotopically labeled P, N, and carbon with a flax–sorghum CMN, the C_3_ flax was found to realize the largest benefit compared with the C_4_ sorghum, investing little, but gaining the most P and nitrogen provided by the CMN (Walder et al. 2012).

Less is known about how AM colonization and connections to a CMN affect intraspecific competition. Most studies examined the consequences for unequal competition between different-sized individuals. Some studies focused on CMNs and their effect on seedling growth of the same species: larger individuals benefit at the expense of smaller neighbors (Janoušková et al. 2011; Weremijewicz and Janos 2013). Large *Andropogon gerardii* plants in the sun received more mineral nutrients than smaller, shaded conspecifics, leading to asymmetric belowground competition (Weremijewicz et al. 2016). These studies looked at the costs and benefits of an individual plant compared to a neighbor within a CMN. However, effects might scale-up beyond the individual plant to influence population-level yields (McGale et al. 2020). Furthermore, using different-sized plants with different growth traits or even different species will complicate the assessment of mycorrhizal effects.

A major limitation of previous work has been the sole reliance on glasshouse studies to experimentally control AM colonization. The glasshouse enables comparisons between inoculated and non-inoculated plants and the ability to separate roots of different plants, but it has numerous disadvantages for the analysis of this highly context-dependent interaction (Bennett and Groten 2022). First, plants are commonly grown in pots in glasshouse studies where they rapidly become pot-bound, unable to freely forage for nutrients, and unnaturally increase competition and other root–root interactions. Moreover, many studies use only a single fungal species for inoculation (Koricheva et al. 2009; Hoeksema et al. 2010). Plants grown in nature can usually explore a much larger soil volume with fewer direct root–root interactions (e.g., see root biomass of glasshouse and field-grown plants in Groten et al. 2015), and roots interact with a high diversity of different AM fungal species. For most field experiments, non-inoculated controls are either missing or confounded by the treatments used to kill the fungal partner (fungicidal fumigation, heat treatment) which alter nutrient or microbiome compositions in addition to the AM fungal association. This problem can be solved using (near)isogenic lines with different capacities to interact with AM fungi, and some have been used in a paired plant in a pot comparison (Facelli et al. 2014; Groten et al. 2015; Bowles et al. 2016; Fabiańska et al. 2020), but rarely have these pot-bound approaches been scaled-up to rigorously test the effects of AM fungi under natural conditions.

Here we elucidate the importance of AM fungi for individual plants connected or not connected by a CMN to neighboring plants of the same species. We hypothesized that: (i) AM increase plant–plant competition so that size inequalities would be amplified when plants differ in responsiveness to AM fungi, and (ii) the presence of non-mycorrhizal plants of the same species would change the growth and fitness outcomes for populations. We used a wild tobacco species, *Nicotiana attenuata*, as a model plant, because its natural history, as well as its interaction with bacterial and fungal microbes, is well-described (Santhanam et al. 2014, 2015; Groten et al. 2015; Schuman and Baldwin 2016). After wildfires, the plants naturally occur in large monocultures (Lynds and Baldwin 1998) of different accessions differing in their chemical composition (Kallenbach et al. 2012; Li et al. 2016) and their capacity to interact with AM fungi (Wang et al. 2018b); hence, intraspecific competition is very high.

This system allows us to study the effect of AM within one species under natural conditions. We established microcosms in the glasshouse (Song et al. 2019) and small plant communities in the plant’s natural habitat where plants differed in their capacity to create a CMN. *Nicotiana attenuata* plants impaired in the interaction with AM fungi due to the RNAi silencing of a calcium- and calmodulin-dependent protein kinase (Groten et al. 2015; Wang et al. 2018b) were compared against isogenic transformation control plants using a fully functional empty vector (EV). The RNAi silenced plants harbor only a single insert, and three independently transformed lines were evaluated in the glasshouse and field in an earlier study giving similar results (Groten et al. 2015). Plants were grown either in initially size-matched monoculture communities or communities consisting of the two lines. We measured growth (rosette size, stalk length, branch lengths and numbers, photosynthesis and nutrient contents) and fitness parameters (flower, capsule, and seed numbers) of plants from the three community types in the glasshouse and field. While individual plants were affected by capacity of their neighbors to interact with AM, growth performance at community level was similar for monocultures and mixed cultures.

## Materials and methods

### Plant material and growth conditions

#### Plant information and germination

The two transgenic lines were generated from a 31st generation wild-type inbred line of *Nicotiana* *attenuata* originating from a seed collection at the DI Ranch (Santa Clara, UT) in southwestern USA in 1988. For all experiments, diploid homozygotes of the second generation of inverted repeat (ir) calcium- and calmodulin-dependent protein kinase (CCaMK, A09-1212-1; Groten et al. 2015) and a pSOL3 empty vector (EV, A-04–266-3; Bubner et al. 2006) as control, each harboring a single transgene insertion were used. The ir*CCaMK* line does not show any internal colonization structures by AM fungal, but exhibits a highly similar vegetative growth when compared to WT plants in absence of AM fungi (Groten et al. 2015). In this previous study, three independently transformed lines of irCCaMK plants were phenotyped under field and glasshouse conditions and all three showed a similar phenotype. Line A09-1212-1 has been extensively used in several transcriptional and metabolomics phenotyping experiments (Wang et al. 2018a, b; Song et al. 2019) and all evidence is consistent with the inference that the plant’s phenotypes arise solely from the silencing of the targeted CCaMK. pSOL3NC, the selectable marker of the EV line, does not result in off-target effects. Thus, growth and other traits of the EV line have been shown to be equivalent to the WT inbred line over several years of field studies (Kessler et al. 2008). Seeds of both transgenic lines were sterilized and germinated according to Krugel et al. (2002).

#### Experimental setups

**Fig. 1:**
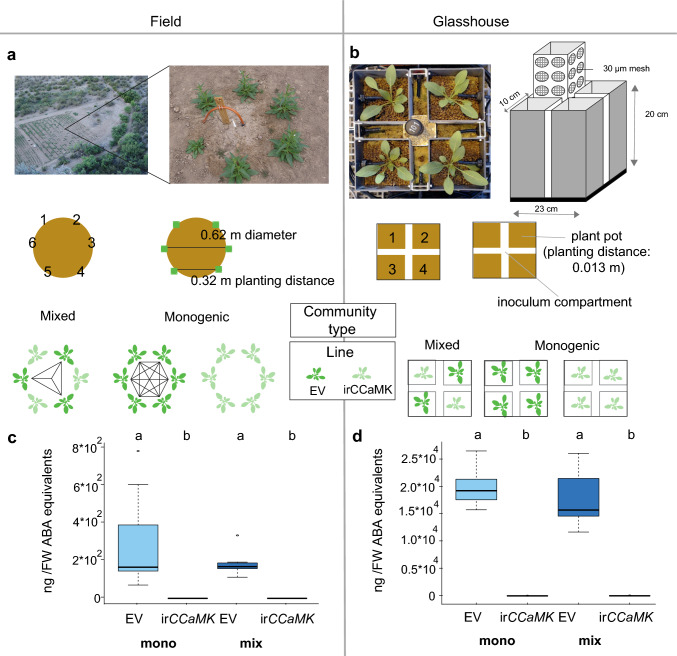
Experimental setup under field and glasshouse conditions and blumenol C levels as markers of arbuscular root colonization. Plant community system to investigate the effect of AM fungi on the intraspecific competitive ability of *Nicotiana attenuata* plants in the field (**a**) and glasshouse (**b**) experiments. Plants were arranged in groups of six in the field and four in the glasshouse and irrigated by a drip line system. In the glasshouse, plants were separated by a cross-shaped fungal compartment. Plant pot walls facing the AM fungal (inoculum) compartment contained 12 openings covered by a 30 μm mesh preventing root–root contact, but allowing penetration by hyphae. An empty vector (EV) and an isogenic line, impaired in the interaction with AM by silencing of *CCaMK* (ir*CCamK*)*,* were used to generate three communities with different mycorrhizal connectivity for experiments in field and glasshouse. **c**, **d** 11-Carboxyblumenol C glucoside contents in roots of the different communities as proxy of root arbuscule colonization (*n* = 7 per group). Peak areas were normalized to the internal standard D6-ABA and results are shown as ABA equivalents. Roots were harvested at the end of the experiment. Different letters indicate significant differences among the different genotype–community combinations (Statistics: Dunn’s Kruskal–Wallis multiple comparison *P* values adjusted with the Benjamini–Hochberg method, *P* < 0.01)

To study the importance of AM for plants in nature, we used two transgenic lines, one impaired (ir*CCaMK*) and another fully capable of mycorrhizal colonization (EV), and created communities with different capacities to interact with AM fungi. Experiments with matching plant communities were conducted in a field plot at the Lytle Ranch Preserve, in SW Utah, located in the Great Basin Desert USA (latitude 37.146, longitude 114.020) and in the institute’s glasshouse in Jena, Germany (Fig. 1a–d). One month prior to planting during each field season, the field plot was plowed and harrowed in six directions to randomize the soil conditions across the plot. Seeds of transformed *N.* *attenuata* lines (ir*CCaMK* and EV) were imported and released under the US Department of Agricultural Animal and Plant Health Inspection Service (APHIS) notification numbers 07-341-101 m (import EV), 10-004-105 m (import CCaMK), 13-350-101r (release in 2014), and 16-013-102r (release in 2016).

#### Field experiments

We performed two field experiments by arranging plants into communities of six plants each grown in circles (Fig. 1a). As the field plot is located in the plant’s native habitat and the experiments timed with the native populations’ phenology, the experiments fully reflect natural conditions (Lynds and Baldwin 1998). The experiments were conducted in April–June in 2014 (Experiment 1) and repeated in 2016 (Experiment 2). The two experiments produced similar results, but not all parameters were measured during both years; hence, some results are only shown for a single year.

For their gradual adaption to high sunlight and low relative humidity, Petri-dish-germinated seedlings were transferred into 50 mm peat pellets (Jiffy 703, Always Grows, Sandusky, OH, USA, http://www.alwaysgrows.com/) previously hydrated in Borax (1:100 dilution of a 1.1 g L^−1^ stock solution), 14 days after germination. These seedlings were kept under shaded conditions in closed 38-quart plastic shoeboxes with transparent lids which were floated on basins of continuously flowing water to maintain temperatures in the 15–30 °C range (Wang et al. 2018a). In 2016, the system was simplified and Jiffy pots were hydrated with borax supplemented with soil microbes from a natural *N. attenuata* population along the Eardly road (latitude 37.101880, longitude 113.975212). The soil solution (5 g L^−1^) was stirred with a drill-operated mixer for 5 min, allowed to settle for 30 min, and used to hydrate Jiffies at 1L/100 jiffies. After 2 weeks of additional environmental adaption (opened boxes in insect-proof tents), size-matched seedlings were planted into communities of six plants, and irrigated until roots were established. In 2014, plants were irrigated through a hose that was placed in the center of the plant community. In 2016, a drip irrigation system was used, providing a dripper for each plant (Fig. 1).

Communities were organized in circles of 0.62 m diameters resulting in a between-plant planting distance of 0.32 m (Fig. 1). Three different communities, one mixed community (Mix-EV and -ir*CCaMK*) and two monogenic communities (Mono-EV/ir*CCaMK*), were created (Fig. 1c). Mixed communities contained an equal number of plants for each transgenic line. To randomize spatial variation in soil quality, including the AM fungal distribution and biotic factors, a fixed number of communities was arranged in blocks over the field plot. In 2016, each block consisted of two monogenic, one of each type (Mono-EV and -ir*CCaMK*), and two mixed communities, and the total number of blocks was 10; hence, 120 total plants per line were used. In 2014, each block consisted of two monogenic and one mixed community (total 12 blocks). 280 g of superphosphate (Star Nursery, Utah, USA) were mixed with the soil surrounding the hose before planting. Additional fertilizers (borax 1.5 g; ammonium sulfate 180 g; potassium nitrate 80 g) and smoke solution (House of Herbs, Passaic, NJ, USA) were added in solution (40 mL diluted in 1.5 L of water) through the hose. Water was pumped through the irrigation system before planting. In 2016, no fertilizer was added to enhance the AM fungal–plant interactions.

#### Glasshouse experiments

In the field, root–root contact (direct competition) cannot be excluded and the spatial distribution of AM fungi and soil nutrients is not predictable. The glasshouse experiment was carried out from April until the end of June 2016. Size-matched *N.* *attenuata* seedlings were planted into communities 14 days after germination and covered with transparent plastic cups for 4 days to adapt the seedlings to the lower relative humidity of the glasshouse. As described for the field experiments, the same two transgenic lines (EV and ir*CCaMK*) were organized into three different communities (Fig. 1d) in a microcosm system with four plants (Fig. 1b). In contrast to the field, the 4 plants in the same pot were separated with a cross-shaped compartment with 12 openings covered by a 30 μm mesh ruling out root–root contact, but allowing the penetration of AM fungal hyphae (Song et al. 2019; Fig. 1). In total, 40 sets of 4-plant-communities were established (20 mixed, and 10 of each monoculture).

Plant pots (size per pot = 10 L × 10 W × 20 H cm) with a volume of 2 L each were filled with a sterile substrate mixture (1:6:3) of finely ground clay, fine (size: 0.4–0. mm) and coarse sand (size: 0.7–1.2 mm). The substrate mixture was autoclaved twice at 121 °C for 30 min beforehand. The same substrate mixture was used for the cross-shaped compartment (space-size = 10 L × 3 W × 20 H cm, core-size = 3 L × 3 W × 20 L cm) with a volume of 2.58 L, but the top 8 cm were replaced with an AM fungal inoculum (~ 1 L per compartment). For the inoculum, soil and chopped leek (*Allium porrum*) roots of a mycorrhizal culture (6th generation) originating from native soil collected from the same field plot at the Lytle Ranch Preserve where the field experiments were done (Wang et al. 2018b) were mixed with the above-mentioned substrate mixture (1:3, v/v). The composition of fungal species (*Funneliformis mosseae* and *Rhizophagus irregularis*) in the inoculum was similar to the composition in native soil (Groten et al. 2015; Wang et al. 2018b).

To establish an AM network in the microcosm system, *Plantago lanceolata* plants were grown in the spaces of the cross-shaped compartment for 4 weeks prior to the start of the experiment. To prevent interplant competition, shoots and the first 1 cm of the root of *Plantago* plants were removed from the cross with the beginning of planting *N.* *attenuata* seedlings in the four pots. This means, AM fungal-colonized *Plantago* roots remained in the core space, but were not in physical contact with the newly planted *N. attenuata* seedlings. During the first week after planting, plants were irrigated with distilled water, and subsequently with a hydroponics solution containing only 1/10 of the regular P_i_-concentration (0.05 mM) using a drip line system (Fig. 1d). During the elongation stage, the P_i_ concentration was increased to 1/4 (0.125 mM) (Groten et al. 2015). Plant pots and the four spaces of the cross-shaped compartment between the pots were watered daily with 56 mL each, for a total volume of 448 mL per pot system. Plants were maintained at standard glasshouse conditions with 16 h of light at 24–28 °C, and 8 h of dark at 20–24 °C and 45–55% humidity.

#### Plant harvest and determination of leaf water content

Plants were carefully removed from soil [field samples, 46 days post-planting (dpp)] or pots (glasshouse samples, 76 dpp). If necessary, running tap water was used to facilitate the process. Shoots were cut at root/shoot junctions and weighed. The third through the fifth stem leaf were excised, separately weighed and dried either at ambient temperatures for 3 days (field samples: 22–40 °C, < 10% rh) or at 72 °C in a drying oven for 2 days (glasshouse samples). After drying, leaves were re-weighed to determine water contents.

### Quantification of plant growth performance, measurement of photosynthesis and fitness parameters

To assess plant growth and fitness, a spectrum of measurements was taken during generative and reproductive phases of growth. In the early stages of rosette growth, 20–25 dpp, images were taken of each plant together with a 9 cm × 9 cm sheet of paper as a scale. For the analysis of rosette diameters and leaf area, pictures were converted to grayscale after adjustment of the color threshold with ImageJ (version 1.51n). A 1 cm scale was used to define the number of pixels per cm. Under Set Measurements, options for Feret’s diameter and Area were chosen to calculate rosette diameter and leaf area. At the beginning of reproductive stalk elongation (25 dpp), stalk lengths were recorded every third day (glasshouse experiment) or once a week (field experiment) for 5–6 weeks. In the field, photosynthetic measurements were conducted at an early stage of elongation (20–25 dpp), measuring the first or second stem leaf. Using the LI-6400XT portable photosynthesis analysis system (Li-Cor Bioscience, Lincoln, NE, USA), net carbon assimilation rates (Ac) were measured at different CO_2_ concentrations (50, 200, 400, 600, 800, 1000, 1200, 1400 µmol m^−2^ s^−1^). All measurements were taken during a cloudless day from 8 am to 12 pm under constant airflow (500 µmol s^−1^), light intensity (2000 µmol m^−2^), and block temperature (25 °C) conditions. In the glasshouse, relative chlorophyll contents were determined with a leaf chlorophyll meter (SPAD-502Plus, Konica Minolta) (66 dpp). During reproductive growth, fully open flowers were counted every third day for 4–5 weeks. In the field, capsules were removed before seed dehiscence and counted when stems turned dry (50–60 dpp). Under glasshouse conditions, seed capsules were counted without removal (45–65 dpp). Seed numbers per capsules, collected from branches of the same lengths, were counted with ImageJ following the same procedure as described above, but using the Analyze Particles instead of the Set Measurement option.

### Quantification of 11-carboxyblumenol-C-glucoside levels as proxies of AM colonization rates

Blumenols were extracted following previously described procedures (Wang et al. 2018a; Mindt et al. 2019). Root samples were ground in liquid nitrogen. 100 mg of plant tissue was extracted with 800 mL of 80% MeOH including 10 ng stable isotope-labeled abscisic acid (D6-ABA, HPC Standards GmbH, Borsdorf, Germany) as an internal standard. After grinding for 60 s at 1100 strokes in a Genogrinder, samples were centrifuged for 20 min at 4 °C and the supernatant was collected and analyzed with a Bruker Elite EvoQ triple quadrupole MS equipped with a heated electrospray ionization ion source as described previously (Mindt et al. 2019). Roots showed high blumenol levels indicative of arbuscular root colonization in EV plants but not in ir*CCaMK* plants, with about 80–100 times higher values in the glasshouse compared to the field at the time of final harvest (Fig. 1c, d).

### Determination of leaf and soil nutrient contents

We determined leaf nutrient contents for field experiment 1 and soil nutrient contents for field experiment 2. The 3rd–5th stem leaf was harvested and stored in paper bags. Soil samples were excavated directly from the rooting zone, 15 cm from the stem to depth of 15 cm. Leaf and soil materials were dried for 3 days in a covered box to avoid direct sunlight, at temperatures approaching ~ 60 °C. For grinding, mixing and homogenizing a mixer mill MM 400 was used. Leaf and soil material was ground at 30 Hz three times for 30 s. Sample aliquoting and measurement of carbon and minerals were done by the Laboratory for Spectrometry at the Max Planck Institute for Biogeochemistry in Jena (Germany) using an elemental analyzer and inductively coupled plasma (ICP)-Atomic Emission Spectrometer Optima 3300 DV (PerkinElmer).

### Statistical analyses

To compare response variables (stalk length, flower number, etc.) between different communities including random (blocks) and fixed effects (time), we used a mixed-effect model approach. Linear (LMER) and generalized linear mixed-effects models (GLMER) were generated according to the experimental design and evaluated with Akaike information criterion (AIC). The best models were tested using estimated marginal means (EMMEANS) with incorporated support for (G)LMER and Tukey adjusted comparisons. An alpha-value of 0.05 was used as a cutoff for significant differences. Statistical analyses were performed with R studio (version 2022.02.3 Build 554) and the program R version 4.2.1 (Team 2016).

## Results

### AM-harboring plants (EV) outcompete neighboring isogenic lines impaired in AM interactions

When grown as individual plants, EV and ir*CCaMK* plants did not differ in shoot and root biomass in the field and glasshouse (Groten et al. 2015). However, in nature, wild tobacco plants often grow under intense intraspecific competition in monoculture populations that occur after wild fires (Lynds and Baldwin 1998). To evaluate if harboring an AM symbiosis influences these plant–plant interactions, we quantified different growth parameters as measures of competitive ability.

**Fig. 2:**
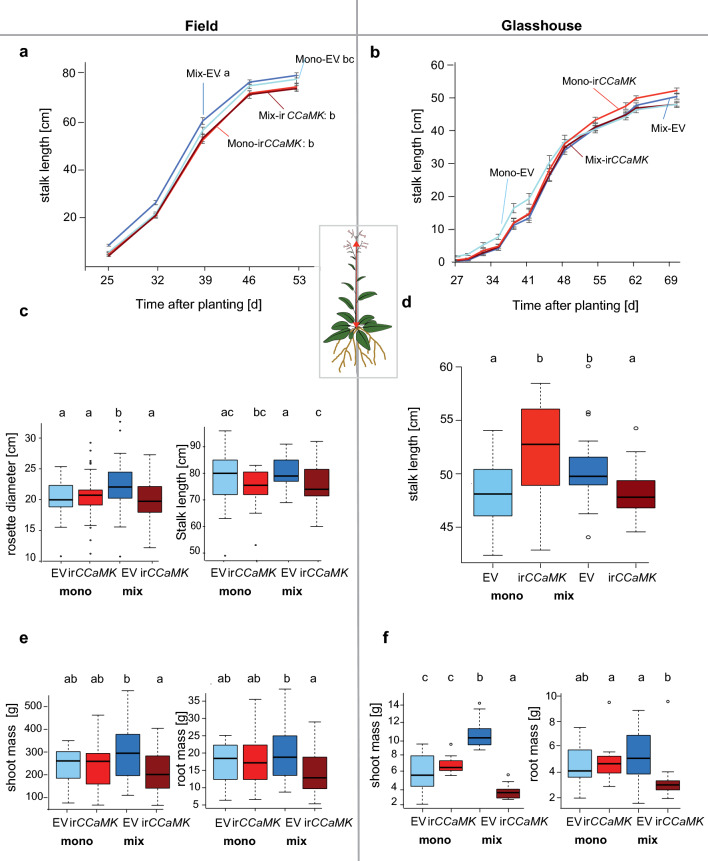
In the field and glasshouse, EV and ir*CCaMK* plants grown in competition differed significantly in stalk length and in biomass with lower values for plants impaired in AM fungal interactions grown in competition with fully functional EV plants. Stalk length growth over time for different communities of plants grown in the field (**a**: Field Exp. 2, *n* = 27–37 per group per time point) and glasshouse (**b**: *n* = 20 per group per time point). Stalk length at final harvest time for different communities of plants grown in the field (**c**: Field Exp. 2, *n* = 27–37 per group) and glasshouse (**d**: *n* = 20 per group). Shoot and root fresh weights for different communities of plants grown in the field. **e** Field Exp. 1 (*n* = 24–33 per group). **f** Glasshouse (*n* = 20 per group). Different letters indicate significant differences among the different genotype–community combinations (for panels **a** and **b**, letters follow line labels). Statistics: EMMEANS with incorporated support for (G)LMER and Tukey adjustment, *P* < 0.05

In the field, we observed significant changes in the growth of stalks over time between EV and ir*CCaMK* plants of mixed, but not for EV and ir*CCaMK* plants of monogenic communities (Fig. 2a). By harvest time, EV plants in mixed communities were significantly taller than ir*CCaMK* plants (46 dpp, Fig. 2c). We obtained similar results in an independent field experiment (Suppl. Fig. S1a, b). Total leaf areas were significantly larger for EV plants of mixed communities compared to those of ir*CCaMK* plants (Suppl. Fig. S2a), while rosette sizes did not differ consistently for the two independent field experiments (Suppl. Fig. S1a and Suppl. Fig. S2c). Consistent with the observed differences in stalk lengths, EV plants of mixed communities had significantly larger shoot and root biomasses than did ir*CCaMK* plants of the same community type in the field, while plants of monogenic communities (EV or ir*CCaMK*) did not differ (Fig. 2e).

In the glasshouse, we also found higher shoot and root biomasses of EV grown in mixed communities compared to EV and ir*CCaMK* plants of monogenic communities (Fig. 2f). AM fungal-colonized EV plants of mixed communities had up to threefold greater shoot and twofold greater root biomasses in comparison to ir*CCaMK* plants of the same community type (Fig. 2f). However, growth differences between the genotypes only became obvious at the later stages of growth in the glasshouse (Fig. 2b, d). Around 50 dpp, EV plants of mixed communities began to grow taller than ir*CCaMK* plants, and at 62 dpp, they had considerably outperformed ir*CCaMK* plants (Fig. 2b).

### Monocultures show similar growth parameters independent of their mycorrhizal status

In contrast to those of mixed communities, plants of monogenic communities did not differ in total leaf area, stalk length, and biomass in the field (Suppl. Fig. S1d, Fig. 2c, e). Surprisingly, in the glasshouse, ir*CCaMK* plants of monogenic communities grew significantly taller than EV plants of monogenic communities (Fig. 2d), while biomasses did not differ among monocultures (Fig. 2f). In contrast to the field, in the glasshouse, EV plants of mixed communities had significantly longer branch lengths and higher branch numbers than ir*CCaMK* plants (Suppl. Fig. S2c, d). On average, they were 11 cm longer and had two more branches. We found no significant differences for branch length and numbers between plants of the monogenic communities.

### Chlorophyll contents and photosynthesis rates are only altered when AM- and non-AM plants compete, while nutrient levels of field-grown plants depend on the AM status

Major drivers of plant productivity are nutrient acquisition and photosynthesis. To evaluate if photosynthetic rates are influenced by AM interactions, we measured CO_2_ assimilation (A) vs. intercellular CO_2_ concentration (Ci) of plants in the different communities. Results from Field Exp. 1 revealed that the A/Ci curves of EV plants of mixed communities and also EV and ir*CCaMK* plants of monogenic communities were higher than those of ir*CCaMK* plants of mixed communities (Suppl. Fig. S3a). Chlorophyll contents of glasshouse-grown plants followed the same pattern. Leaves of EV plants in mixed, as well as EV and ir*CCaMK* plants of monogenic communities exhibited higher chlorophyll contents than leaves of ir*CCaMK* plants of mixed communities, while chlorophyll contents among plants of monogenic communities did not vary (Suppl. Fig. S3b). Interestingly, in mixed communities, leaf water contents of EV plants tended to be higher than those of ir*CCaMK* plants in both field experiments, but differences were not significant (Suppl. Fig. S2b, Suppl. Fig. S1d).

Independent of the community, we found significantly higher levels of foliar nitrogen (N), and phosphorous (P) of AM fungal colonized (EV) than of non-colonized (ir*CCaMK*) plants (Suppl. Fig. S4a, b). Also total carbon, copper, and potassium contents in leaves were significantly different between monogenic communities, with EV plants having higher levels than ir*CCaMK* plants (Suppl. Fig. S4c-e). While foliar nutrient contents varied among plants of the different communities, nutrient contents (nitrogen, organic and inorganic carbon) in the soil close to the root system did not differ (Suppl. Fig. S5c-e).

From these results, we conclude, that under competitive conditions, AM associations increase plant productivity (i.e., increased biomass), probably due to improved water and chlorophyll contents as well as photosynthetic rates.

### AM colonization increases reproductive performance under competitive conditions

**Fig. 3:**
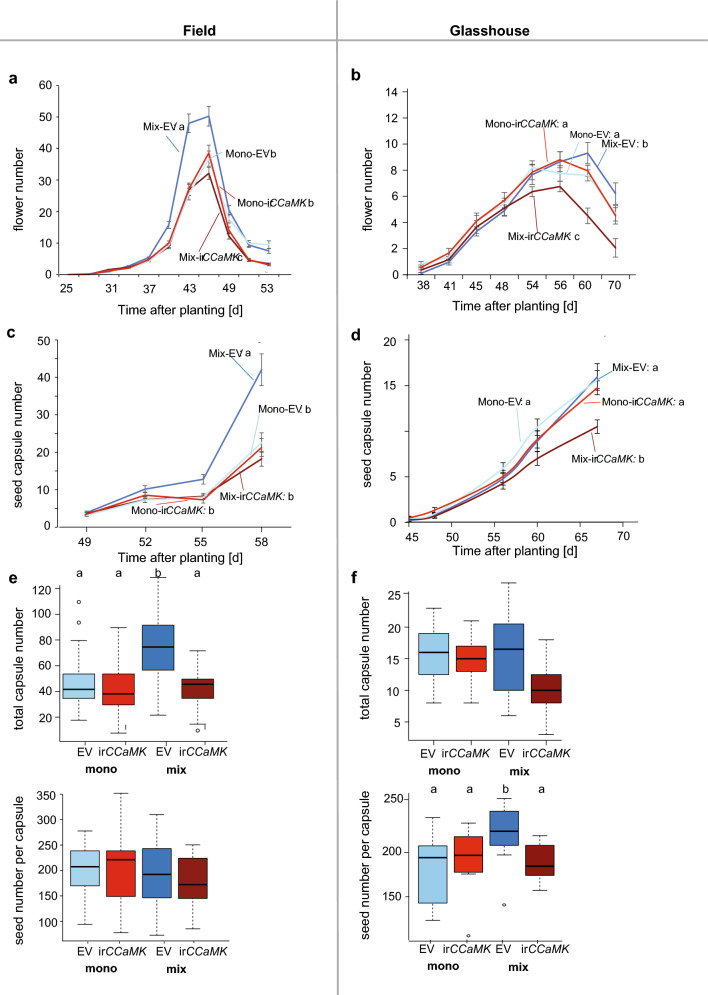
EV plants grown in competition in mixed communities had greater fitness in terms of flower and seed capsules numbers than did irCCaMK plants in both the field and in the glasshouse. Flower number over time of different communities grown in the field (**a**: Exp. 2, *n* = 27–37 per group per time point) and in the glasshouse (**b**: *n* = 20 per group per time point). Seed capsule number over time of the different communities grown in the field (**c**: Exp. 2, *n* = 27–37 per group per time point) and in the glasshouse (**d**: *n* = 20 per group per time point). Total seed capsule number (left) and seed number per capsule (right) of different communities grown in the field (**e**: Exp. 2, left: *n* = 27–37 per group, right: *n* = 16–28 per group) and in the glasshouse (**f**: left: *n* = 20 per group, right: *n* = 12 per group). (Statistics: EMMEANS with incorporated support for (G)LMER and Tukey adjustment, *P* < 0.05). Different letters indicate significant differences among the different genotype–community combinations

To investigate the role of AM fungi in reproductive performance of wild tobacco communities, we recorded flower and capsule numbers, as well as seed numbers per capsule for plants of the different communities. In the field, flower production reached its maximum between 44 and 47 dpp (Fig. 3a). During this period, EV plants in mixed communities produced twice as many flowers as ir*CCaMK* plants of the same community type and thus significantly outperformed ir*CCaMK* plants, while for EV and ir*CCaMK* plants of monogenic communities, flower production rate did not differ (Fig. 3a). In total, EV plants of mixed communities had significantly higher flower numbers compared to ir*CCaMK* plants of mixed communities, as well as EV and ir*CCaMK* plants of monogenic communities (Suppl. Fig. S4a). Consistent with flower production rates, total flower numbers for EV and ir*CCaMK* plants of monogenic communities did not vary (Suppl. Fig. S4a). At the peak of flower production, seed capsules started to grow and around 53 dpp, EV plants of mixed communities had higher seed capsule numbers than did ir*CCaMK* plants of mixed communities, as well as EV and ir*CCaMK* plants of monogenic communities (Fig. 3c). In general, seed capsule numbers over time were significantly different between EV and ir*CCaMK* plants in mixed communities, but did not differ for EV and ir*CCaMK* plants of monogenic communities (Fig. 3c). EV plants of mixed communities produced 80% more seed capsules than did ir*CCaMK* plants of the same community type at 58 dpp (Fig. 3e). While the total seed capsule numbers of EV and ir*CCaMK* plants in mixed communities were significantly different, the total seed capsule numbers for EV and ir*CCaMK* plants of monogenic communities were similar (Fig. 3e). The numbers of seeds per capsule for EV and ir*CCaMK* plants of mixed or monogenic communities did not differ (Fig. 3e).

Flower production in the glasshouse reached its maximum between 54 and 60 dpp, 10–15 days after peak flower production in the field (Fig. 3b). Similar to the field, EV plants of mixed communities had twice as many flowers as ir*CCaMK* plants of the same community type at the time of peak flower production in ir*CCaMK* plants (Fig. 3b). Accordingly, the flower production rate was significantly different for EV and ir*CCaMK* plants in mixed communities, while those of the monogenic communities did not vary (Fig. 3b). Total flower numbers of EV plants of mixed and also of EV and ir*CCaMK* plants of the monogenic communities were significantly higher than those of ir*CCaMK* plants of mixed communities (Suppl. Fig. S6). Seed capsule maturation in the glasshouse started at around the same time as in the field (45 dpp) (Fig. 3d). Results for seed capsule number production over time and total seed capsule numbers followed the same pattern as in the field, with EV plants of mixed communities, as well as EV and ir*CCaMK* plants of monogenic communities being significantly greater than those of ir*CCaMK* plants of mixed communities (Fig. 3d). EV plants of mixed communities produced 50% more seed capsules than did ir*CCaMK* plants of the same community type. The numbers of seeds per capsule were significantly greater for EV than for ir*CCaMK* plants in mixed communities (Fig. 3f).

In summary, AM colonization promotes reproductive growth under competitive conditions. Colonized plants competing with non-colonized plants showed higher numbers of flowers, seed capsules, and seeds per capsule.

### Impact of AM on community-level productivity

In addition to the analysis of individual genotype performance within the different communities, we also examined community-level productivity to evaluate: (i) if AM associations increased the overall fitness of a plant community, and (ii) if heterogeneous communities with different capacities to interact with AM fungi outperformed monogenic communities. AM-specific effects, which were in our setup equivalent to genotypic differences, should be seen as differences between the monogenic communities, while mixed communities would have intermediate values (i.e., Mix, Mono, Mono: ab, a, b). Changes resulting from AM-mediated intraspecific competition should result in differences between mixed and monogenic communities (i.e., Mix, Mono, Mono: a, b, b).

**Fig. 4:**
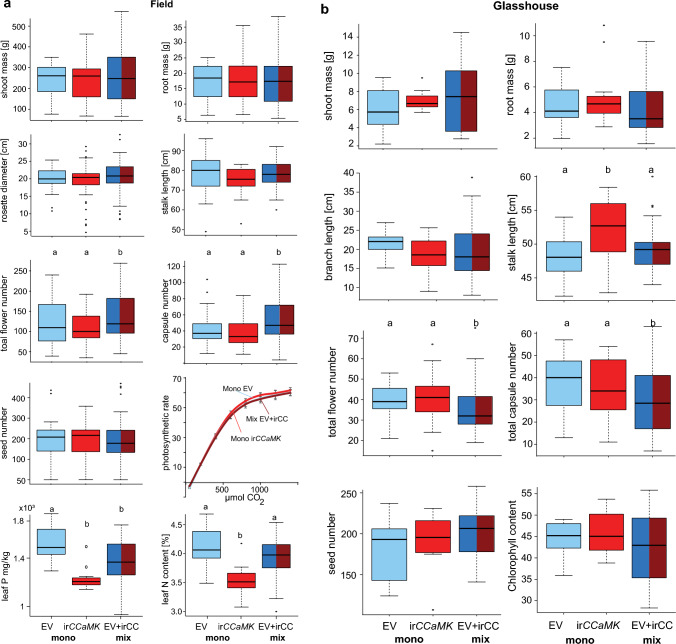
Community-level comparisons of vegetative and generative growth parameters of the field (**a**) and glasshouse (**b**) experiments. Comparisons at community level are based on three different communities: two monogenic (mono-EV and mono-ir*CCaMK*) communities and one mixed community (EV and irCCaMK). Statistics: EMMEANS with incorporated support for (G)LM(ER) and Tukey adjustment, *P* < 0.05. Different letters indicate significant differences among the different genotype–community combinations

Interestingly, we did not observe significant differences in growth among the three community types (Fig. 4b). However, in the field, despite similar vegetative growth for all community types, flower and capsule numbers of mixed communities were significantly higher while we observed the opposite result in the glasshouse (Fig. 4a vs. b). The deficit in N in leaves of irCCaMK monocultures could be compensated in mixed communities in the field, but not for P. Total P content of the community was still significantly lower in mixed communities compared to EV monocultures (Fig. 4a). Hence, we only observed a limited net benefit of AM fungi for plant communities.

## Discussion

We manipulated a plant’s capacity to connect to a mycorrhizal network to evaluate whether AM colonization modulates neighbor competition for nutrients and if an asymmetry in neighbor competition within a community translates into differences in the productivity of plant communities of the same species. We used two isogenic wild tobacco lines to answer these questions: one line was impaired in interacting with AM fungi due to silencing a key gene of the symbiotic pathway, *CCaMK*, while the second line was fully functional. Both were shown previously to have a similar growth when grown in single pots without inoculum, a prerequisite for this type of comparative study (Rillig et al. 2008). Our system mimics the extremes of nature—the same species, either interacting and connecting or not to a CMN. In natural populations, gradients of AM root colonization and plant responses are commonly found (Wang et al. 2018a; Sawers et al. 2017; Savary et al. 2020). For *N. attenuata,* it was shown that two different accessions (Utah, UT and Arizona, AZ) and the resulting crossing population differ in their mycorrhizal colonization and in the accumulation of the arbuscule-marker carboxyblumenol glucoside in the glasshouse and in the field (Wang et al. 2018a), underlining the ecological importance of our study. Here, we used two experimental systems—an experimental field and a microcosm system—to test our hypotheses. In contrast to our previous study (Wang et al. 2018b), we not only focused on two plants growing in the same small pot, but we created small communities. In the field, the roots could forage unconstrained by pot dimensions for nutrients, connect with neighboring plants and they were exposed to the natural AM fungal community. This is important, as population-level responses that reveal how AM affects plant coexistence and community structure can only be fully addressed in the field (Hart et al. 2003). The additional microcosm approach enabled us to rule out direct effects of root–root competition, and we increased the volume of the pots (4 × for a single plant) to have less pot-constrained root growth. Furthermore, neighbor recognition that might be independent of the AM fungal association is minimized due to the root-free inoculum compartment separating the root systems and thus strongly reducing information about their neighbor’s identity and performance based on belowground root exudates and volatiles (Semchenko et al. 2018; Kong et al. 2018). Similarly, specific bacterial communities which may also alter growth performance (Chen et al. 2012; Gfeller et al. 2019) and microRNAs produced by plants that may act as signaling molecules affecting gene expression in other, nearby plants (Betti et al. 2021), are unlikely to be exchanged.

The results revealed that AM colonization modulates the competitive relationship between plants of the same species differing only in their capacity to interact with AM fungi. In both the field and the glasshouse, AM associations led to an asymmetry in growth and fitness of plants connected to a functional network compared to those not connected. ir*CCaMK* plants grown adjacent to an AM-colonized neighbor had significantly smaller roots, shoots and stalk lengths, and a corresponding lower rate of photosynthesis and chlorophyll content. The loss of access to fungal partners in ir*CCaMK* plants was reflected in a performance gain of neighboring fully functional empty vector plants. These results corroborate and extend the findings of a recent pot study growing the same lines together in a single pot (Wang et al. 2018b). In the previous study, growth differences were much more pronounced, most likely due to higher competition in the same pot with highly limited amounts of nutrients, while in the field and in larger pots without direct root–root interactions, competition is more relaxed. In accordance with this assumption, the plants unable to interact with AM fungi and competing with fully functional plants were clearly P starved (Wang et al. 2018b). Similarly, in tomato and *Medicago*, fully functional wild-type plants outcompeted mutant lines that acted as non-AM fungal surrogate hosts when grown in the same pot in the glasshouse (Facelli et al. 2010, 2014). In all these studies, the AM-harboring partner pre-empted limiting resources. The difference in water content was not significant, but there was a tendency for reduced water content in ir*CCaMK* plants within mixed communities. Recently, it was shown that mycorrhizal fungi can transport water to their host plants, even from sources that cannot be accessed by plant roots (Wang et al. 2022). It was estimated that AM may contribute more than 40% of the water transpired by plants (Kakouridis et al. 2022). Hence, the interaction with AM fungi seems to enable plants to extract the limited water resources more efficiently compared to neighboring ir*CCaMK* plants.

In contrast to the mixed cultures, we did not find significant growth and fitness differences among the lines grown in monoculture. This means that as long as the neighbors have the same “handicap” or advantage, the outcome in terms of growth and fitness is similar. The similarity in growth among the two monocultures in the field and a similar result in the glasshouse also indicates that the adjustments in the field setups between the two field seasons do not affect the questions addressed in this study. Furthermore, in contrast to the monocots rice and maize that show *CCaMK* expression in leaves and have a role in antioxidant defense and ABA signaling (Ma et al. 2012; Shi et al. 2012) in *N. attenuata*, CCaMK is not expressed in leaves (see http://nadh.ice.mpg.de/NaDH/, NIATv7_g40298; Brockmöller et al. 2017). The lack of growth differences between the two lines under non-competitive conditions is in accordance with previous results using the same lines in the field and glasshouse (Groten et al. 2015) and corroborates that intraspecific competition only occurs when different isogenic lines compete for nutrients, while we could not find neighbor effects within the same genotype. However, in our setup, we did not vary the number of plants per pot or plant density in the field, meaning that we may have missed a stronger effect of AM fungal colonization on intraspecific competition. A number of studies have shown that intraspecific density modifies a plant’s response to mycorrhizal colonization (e.g., Schroeder-Moreno and Janos 2008; Collins et al. 2010). For example, increasing densities significantly reduced the biomass of individual mycorrhizal *Trifolium subterraneum* plants but the biomass of individual non-mycorrhizal plants was only slightly reduced (Facelli et al. 1999). Increasing intraspecific competition also decreased growth differences in *Plantago lanceolata* (Ayres et al. 2006).

Though we did not find differences in growth and fitness when the two genotypes were grown in monocultures, P, N, K, and Cu levels were significantly higher in EV plants compared to ir*CCaMK* plants in the field. Consistent with the results presented here, fruits of tomato mutants with reduced mycorrhizal colonization (rmc) compared to their wild-type progenitor were also not altered in growth when grown in monocultures or under non-inoculated conditions, but they still differed in nutrient contents (Cavagnaro et al. 2004, 2006). For these experiments, it is not known if the lower nutrient content resulted in further fitness consequences. Higher nutrient levels may, on the one hand, facilitate the production of defense compounds and make the plants more resistant against necrotrophic pathogens, while, on the other hand, leaves and fruits may become more attractive to herbivores and biotrophic pathogens, as shown for AM-colonized tree saplings (Ferlian et al. 2021).

In contrast to our study, field and glasshouse studies with maize plants defective in mycorrhiza-specific Pi transporter Pht1;6 gene and their wild-type progenitor showed lower nutrient levels and less biomass production, not only when plants were competing for nutrients, but also when plants were grown in monocultures (Fabiańska et al. 2020). We can only speculate about these different results, but one hypothesis is that additional signaling pathways that may govern, e.g., P-transporter expression, had already been initiated in the maize mutants. P transport via the direct root pathway is known to be attenuated during mycorrhizal colonization (Smith et al. 2011). Pht1;6 acts downstream of a symbiosis receptor kinase (SYMRK—the mutation found to be responsible for the reduced mycorrhizal colonization in rmc tomatoes; Nair and Bhargava 2012) and of CCaMK (MacLean et al. 2017). Hence, the direct P pathway may have already been altered by contact with AM fungi in the maize mutants.

As plant size and reproduction are often correlated, size inequalities can result in reproductive inequalities (Weiner 1988). Here, we also show that ir*CCaMK* plants in mixed communities were not only smaller but also produced significantly fewer flowers and capsules, and at least in the glasshouse also less seeds per capsule than neighboring EV plants. Being connected to a hyphal network promotes the performance of genotypes interacting with a mycorrhizal partner, thus providing genotype-specific benefits. Considering our findings in the light of the distribution of genotypes within plant populations, the lower number of capsules and seeds per capsule found in the field for ir*CCaMK* plants growing in competition with EV could lead to changes in the composition of the long-term seed bank in the soil and the frequency of the genotypes would be altered. Data on long-term community composition of different accessions of the same species in relation to the efficiency of interacting with AM fungi are not yet available. Studies based on different species provide contradictory results ranging from strong (van der Heijden and Scheublin 2007) to weak (Milkereit et al. 2018) or no effect (Koch et al. 2012) of AM fungi on plant community productivity and composition. In this context, it might be important to consider that *N. attenuata* germinates in nature after fires, and some studies have shown that fire initially reduces the number of AM fungal spores, while more nutrients are available (Hartnett et al. 2004) which may give plants with no or weak AM interactions a competitive chance.

Given the controversy surrounding CMN benefits, we were not only interested in the performance of individual plants compared to their neighbors, but also whether the productivity of a community is altered when individual plants have different capacities to interact with AM fungi. Focusing on growth parameters, we did not find a net benefit for a specific community type. In mixed cultures, the growth depression of ir*CCaMK* plants was compensated by the performance gains of EV plants, so that mixed communities realized no net benefit. An independent study used the same Na-ir*CCaMK* lines for a different question, and they also found that population-level effects were independent of belowground AM fungal interactions (McGale et al. 2020). However, results for fitness traits (total flower and capsule numbers) were not conclusive—we observed a net benefit in the field but a decline in the glasshouse. We can only speculate about these dissimilar results. One assumption is that the strictly limited amounts of nutrients in the glasshouse study impaired further benefits and only allowed for an exploitation of resources at the expense of the neighbor, while in the field, additional resources were mobilized leading to a net benefit. Across most parameters measured, we observed in the field that EV in mixed communities performed significantly better compared to ir*CCaMK* plants and the single genotype communities, while the major differences in the glasshouse were growth depressions and reduced fitness of ir*CCaMK* and over-performance of EV in mixed communities compared to the two monoculture communities. In addition to pot-bound nutrient limitations of the glasshouse, additional root–root interactions in the field cannot be ruled out. Based on our results, we are unable to disentangle if individual plant–plant competition drives the net fitness benefit in the field or if the connection to the CMN plays an additional role.

In conclusion, these experiments highlight the importance of AM fungi in intraspecific plant–plant competition in natural plant communities, allowing AM-harboring plants to outperform non-harboring conspecifics when competing on a small spatial scale. AM associations provided few demonstrable performance benefits at the community level, consistent with recent critical reviews of the data for CMN benefits in forests (Karst et al. 2023). Mycorrhizae may have profound effects on long-term plant population dynamics—altering the genetic contribution of individuals from one generation to the next.

### *Author contribution statement*

KG and ITB conceived and designed the research. ITB prepared both field experiments, FY generated the data of the field experiment in 2016. KG analyzed the data. KG wrote the first version of the manuscript with further contributions from all authors. All authors read and approved the manuscript.

## Supplementary Information

Below is the link to the electronic supplementary material.

Supplementary file1 (PDF 531 KB)

## Data Availability

The datasets generated during the current study are available from the corresponding author on reasonable request.

## Abbreviations

AM: Arbuscular mycorrhiza
dpp: Days post-planting
EV: Empty vector
ir*CCaMK*: Inverted repeat calcium- and calmodulin-dependent protein kinase
CMN: Common mycorrhizal network
