# Supplementary material for: Arbuscular mycorrhizal fungi influence the intraspecific competitive ability of plants under field and glasshouse conditions
Source: Planta. 2023 Aug 3;258(3):60. doi: 10.1007/s00425-023-04214-z (PMC10400695; doi:10.1007/s00425-023-04214-z)
Supplement: Supplementary file 1 — Supplementary file1 (PDF 531 KB) [file 425_2023_4214_MOESM1_ESM.pdf]

Supplemental Figures

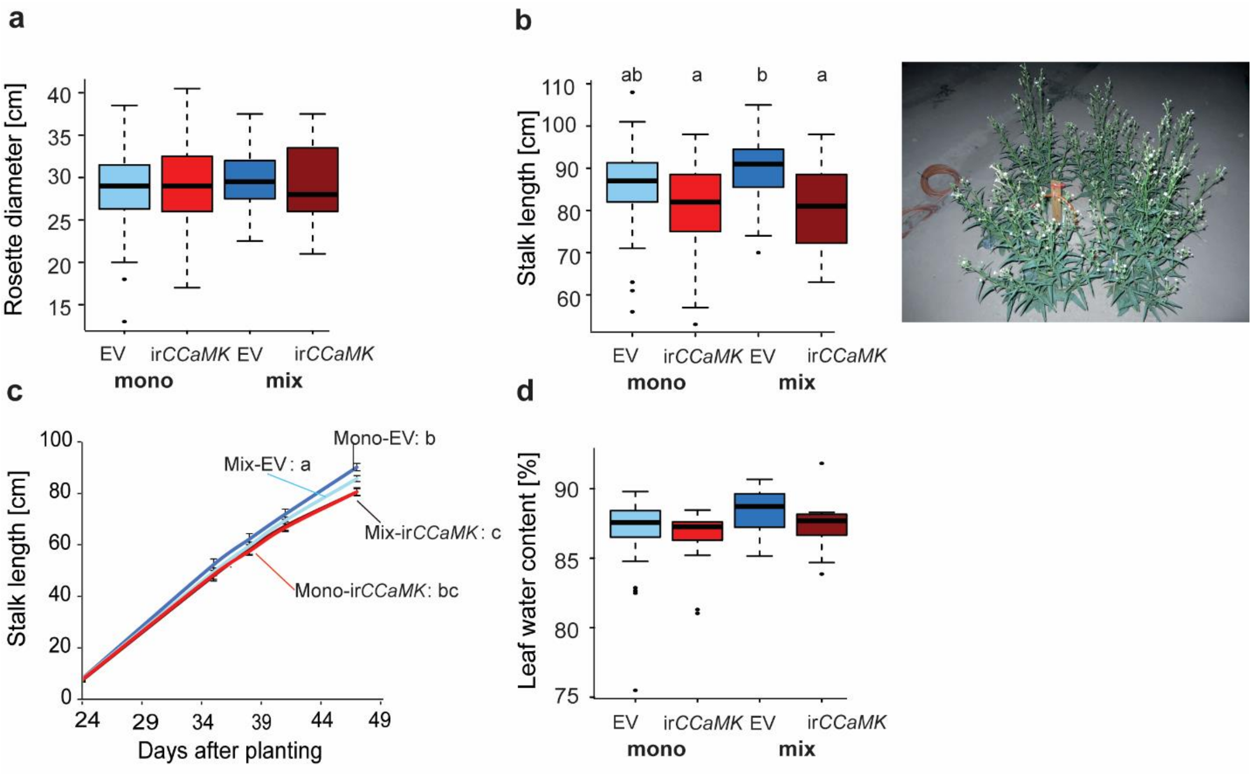

Suppl. Fig. S1: In the field EV plants outperform ir*CCaMK* plants in stalk length. Leaf water contents tended to be lower in ir*CCaMK* plants, but differences were not significant. **a** Rosette diameter (Field Exp. 1, *n*=65-74 per community) and final stalk length for mixed and monogenic communities of plants grown in the field (Field Exp. 1, *n*=64-66 per community). **b** and **c** Mean ± SE stalk length over time of different communities of plants grown in the field (Field Exp. 1, *n*=65-74 per community per time point). Representative picture of a plant community. **d** Leaf water content of different communities of plants grown in the field (Field Exp. 1, N = 30) Statistics were done using EMMEANS with incorporated support for (G)LMER and Tukey adjustment, different letters indicate significant differences, *P*<0.05. In panel **c** letters follow line labels.

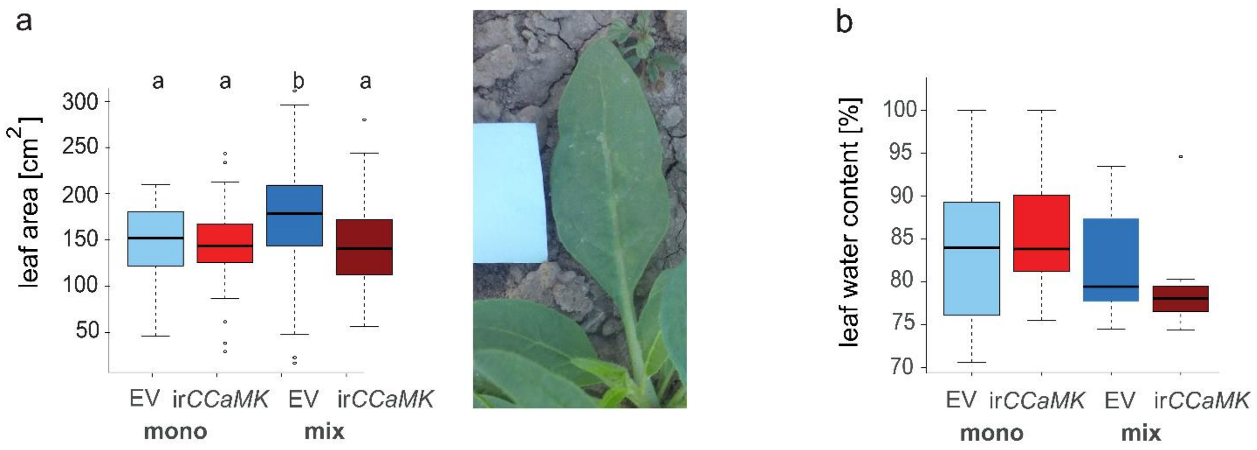

Suppl. Fig. S2: Leaf area (**a**) and water contents (**b**) for mixed and monogenic communities of plants grown in the field (Field Exp. 2, **a** *n*=56-58, **b** *n*=16 per group)**.** Statistics: EMMEANS with incorporated support for (G)LMER and Tukey adjustment, $p<0.05$; different letters indicate significant differences among the different genotype-community combinations.

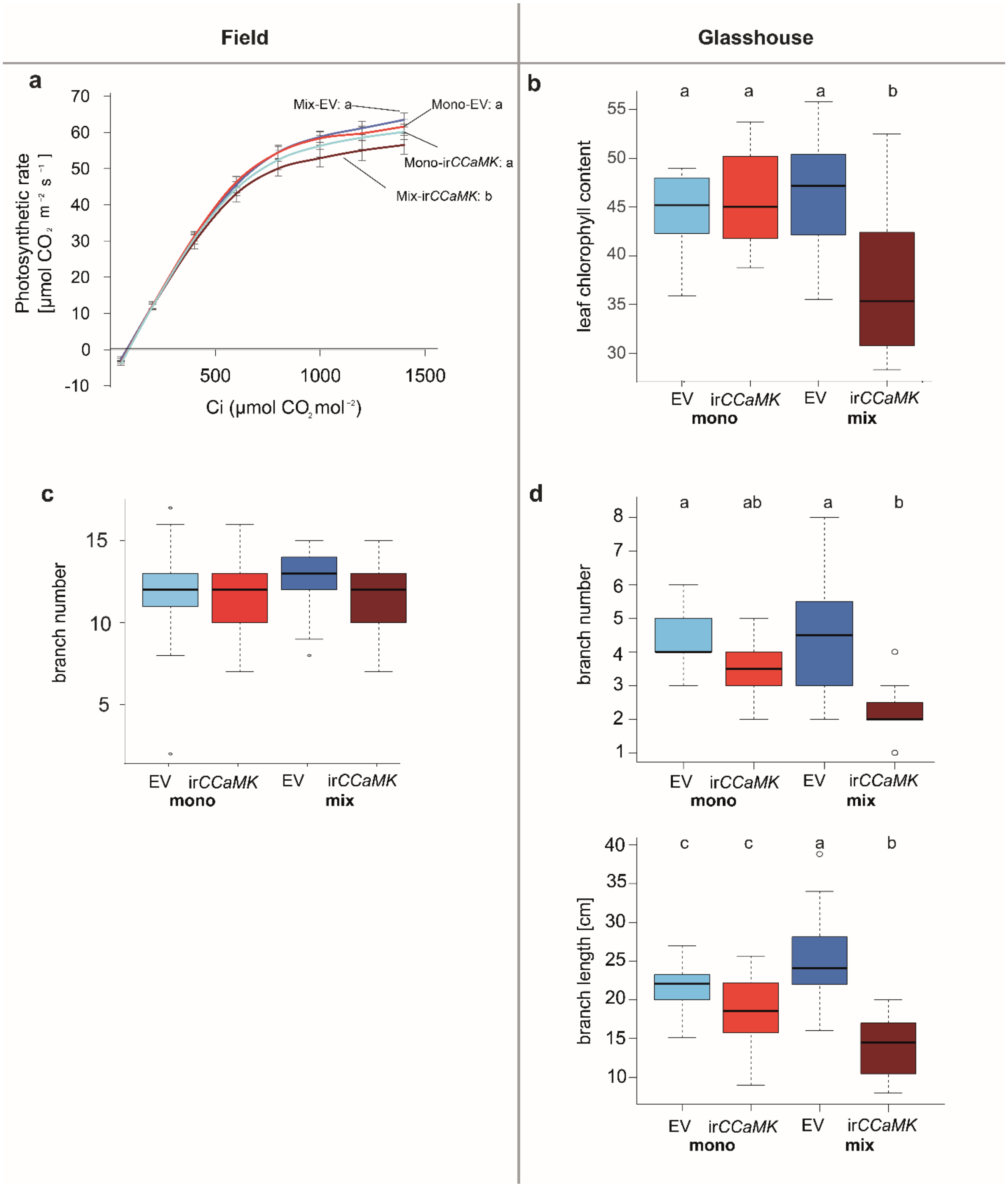

Suppl. Fig. S3: ir*CCaMK* plants grown in mixed communities had a lower photosynthetic rate and chlorophyll content in the field and glasshouse, respectively.

a Mean±SE of photosynthetic rates versus internal leaf CO_2 concentration (Ci) for mixed and monogenic communities of plants grown in the field (Field Exp. 1, n=6 per group). **b** Chlorophyll content of the

second stem leaf for mixed and monogenic communities of plants grown in the glasshouse (*n*=10 per group). **c** and **d** EV plants had significantly more and longer branches than did ir*CCaMK* plants in mixed communities in the glasshouse, but not in the field (Field Exp. 1, N=65-74 per community, Glasshouse, N=20)**.** Different letters indicate significant differences among the different genotype-community combinations: letters in Panel **a** follow line labels. (Statistics: EMMEANS with incorporated support for (G)LMER and Tukey adjustment, $p<0.05$).

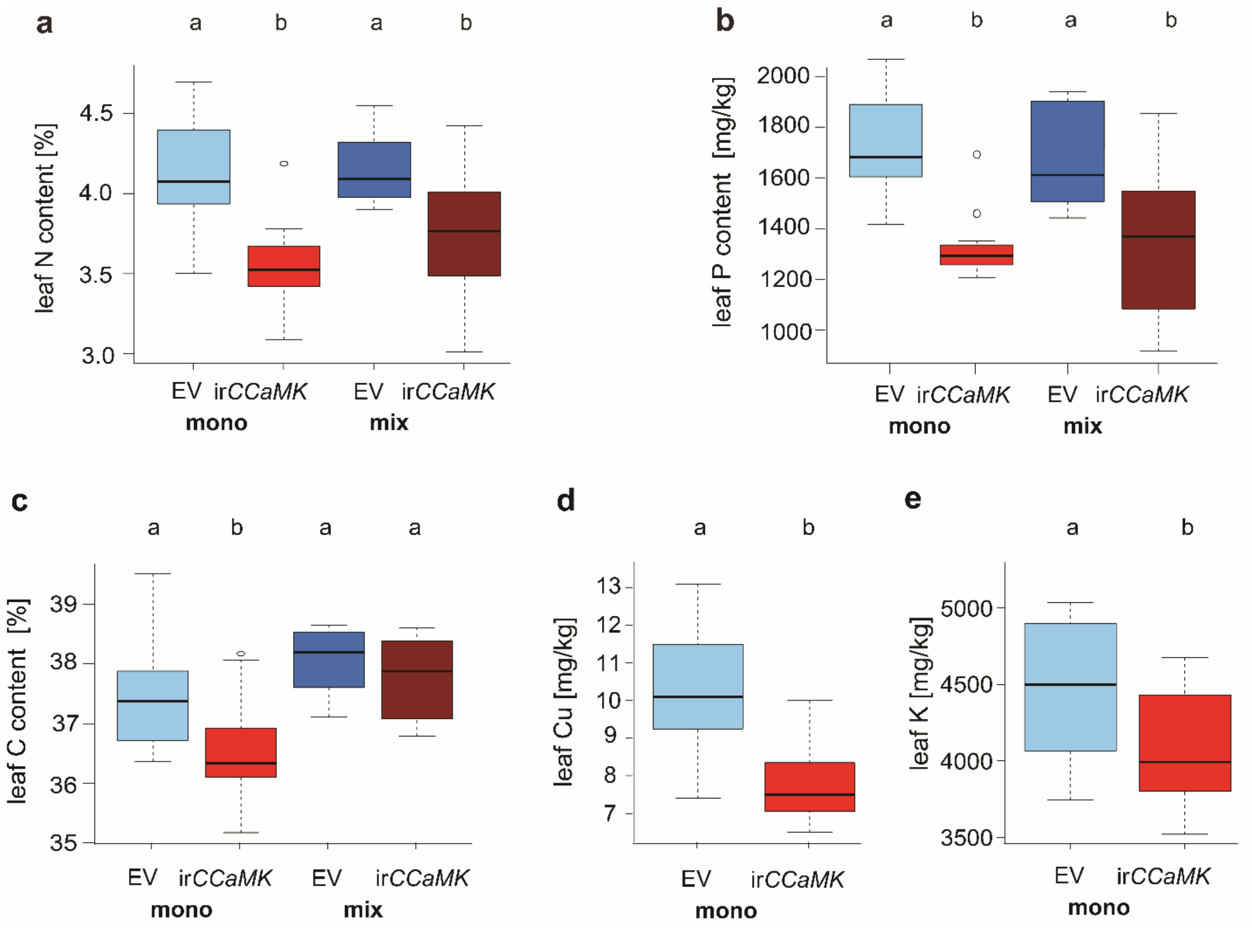

Suppl. Fig. S4: Total leaf nitrogen (N), phosphorus (P) and carbon (C) as well as copper and potassium contents are significantly lower in monocultures of AM-impaired lines.

a Total leaf nitrogen (N), **b** phosphorus (P) , **c** carbon (C), **d** copper (Cu) and **e** potassium (K) contents of 3rd-5th stem leaves for plants of mixed and monogenic communities grown in the field (Field Exp. 1: n=12 per group). Different letters indicate significant differences among the different genotype-community combinations. (Statistics: EMMEANS with incorporated support for (G)LMER and Tukey adjustment, P<0.05).

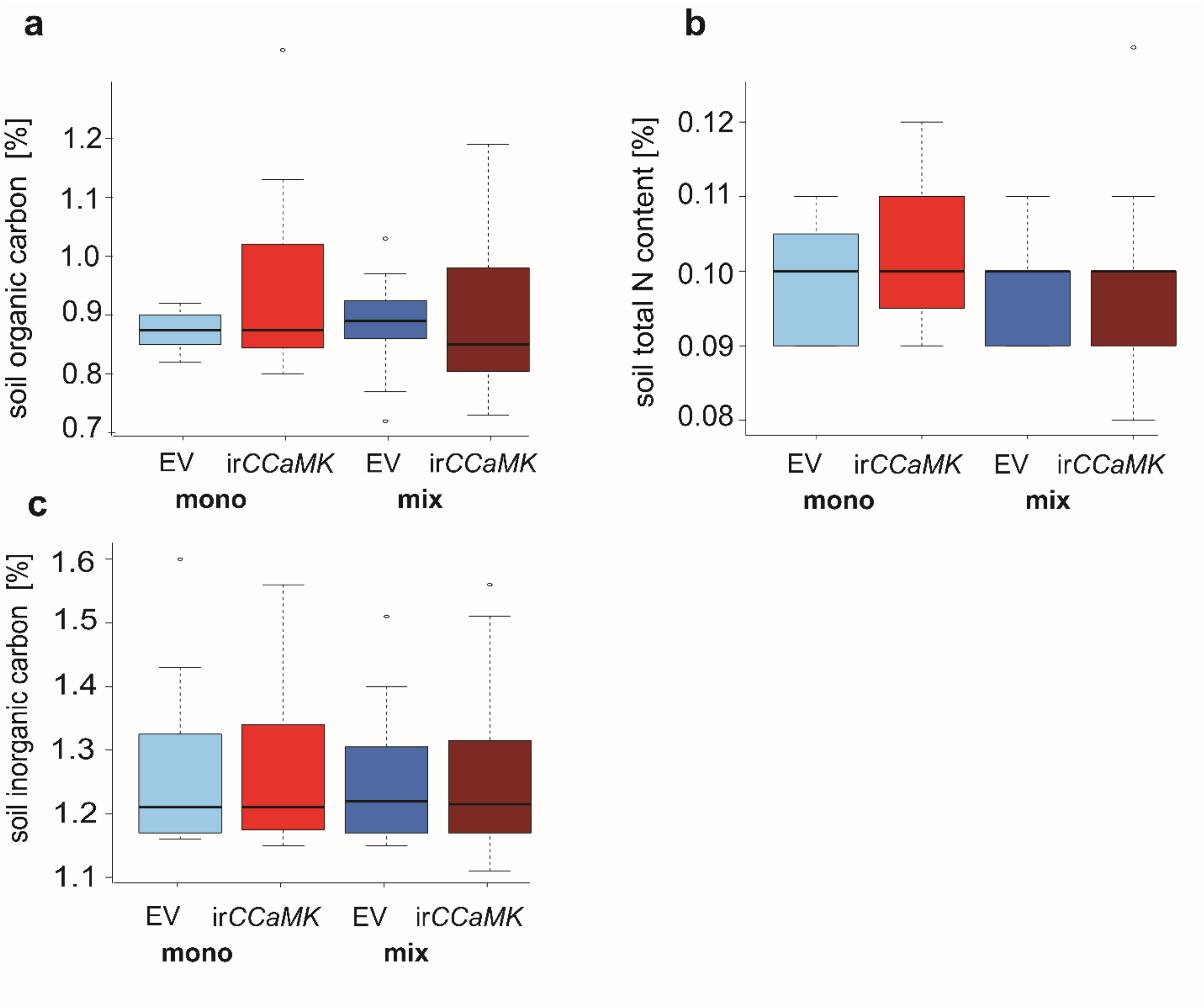

Suppl. Fig. S5: Rhizosphere soil nutrient contents (N, C) do not differ among the communities.

Soil organic carbon (**a**), N (**b**) and inorganic carbon contents collected (**c**) from the rhizosphere of plants grown in the different communities in the field (Field Exp. 2, *n*= 12). Statistics were done using EMMEANS with incorporated support for LME and Tukey adjustment, $P<0.05$.

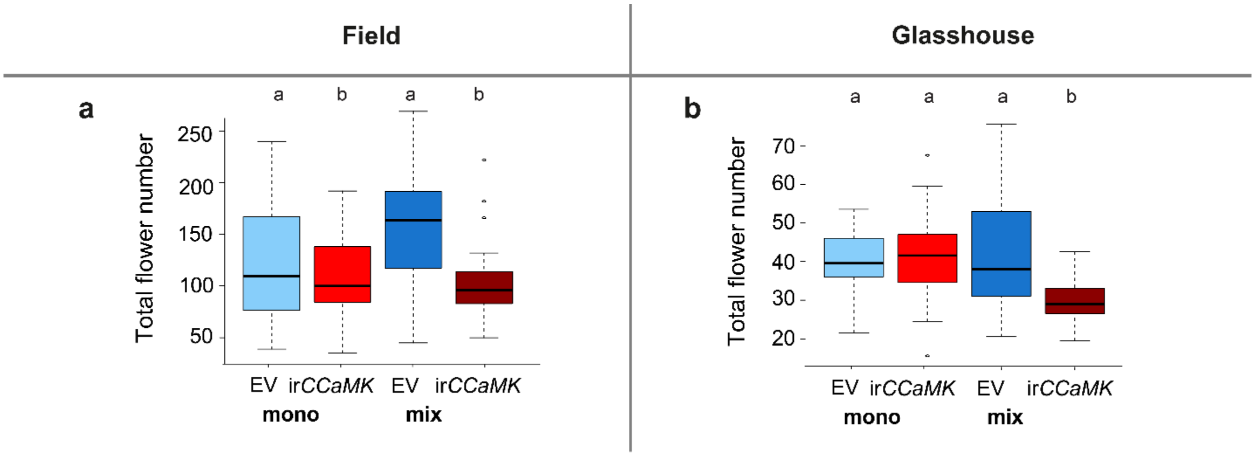

Suppl. Fig. S6: For both field and glasshouse experiments, significant differences were detected between genotypes in mixed communities with EV having considerable higher total flower numbers. **a** and **b** Total flower number per plant in different communities grown in the field (**a**: Field Exp. 2, *n* =25-36) and glasshouse (**b**, *n*=20). Statistics were done using EMMEANS with incorporated support for (G)LMER and Tukey adjustment, *P*<0.05. Different letters indicate significant differences among the different genotype-community combinations.
